# 2,2-[(*E*)-3,3-Diphenyl­prop-2-ene-1,1-di­yl]bis­(3-hy­droxy­cyclo­hex-2-en-1-one)

**DOI:** 10.1107/S1600536811048355

**Published:** 2011-11-23

**Authors:** Jae Kyun Lee, Sun-Joon Min, Yong Seo Cho, Joo Hwan Cha, Hiroyasu Sato

**Affiliations:** a Center for Neuro-Medicine, Korea Institute of Science & Technology, Hwarangro 14-gil, Seongbuk-gu, Seoul 136-791, Republic of Korea; b Advanced Analysis Center, Korea Institute of Science & Technology, Hwarangro 14-gil, Seongbuk-gu, Seoul 136-791, Republic of Korea; c Application Laboratory, Rigaku Corporation, 3-9-12 Matsubara-cho, Akishima-shi, Tokyo 196-8666, Japan

## Abstract

In the title compound, C_27_H_26_O_4_, each of the cyclo­hexenone rings adopts a half-chair conformation. The dihedral angle between the two phenyl rings is 89.53 (5)°. The hy­droxy and carbonyl O atoms face each other and are orientated to allow the formation of two intra­molecular O—H⋯O hydrogen bonds, which are typical of xanthene derivatives.

## Related literature

For the crystal structures of xanthenes derivatives studied recently by our group, see: Cha *et al.* (2011*a* ▶,*b* ▶).
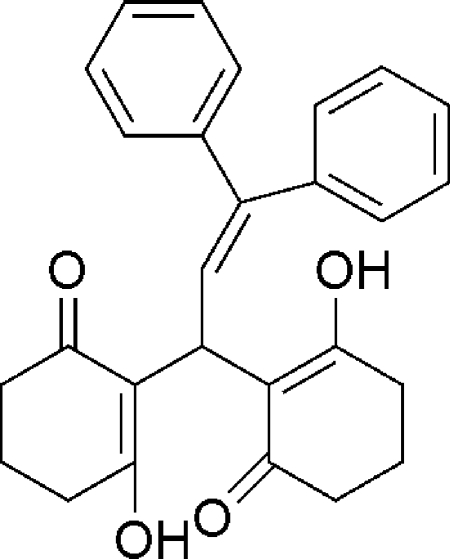


## Experimental

### 

#### Crystal data

C_27_H_26_O_4_
*M* *_r_* = 414.50
Orthorhombic, 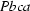

*a* = 9.7329 (5) Å
*b* = 18.6106 (9) Å
*c* = 24.6522 (12) Å
*V* = 4465.4 (4) Å^3^
*Z* = 8
Mo *K*α radiation
μ = 0.08 mm^−1^
*T* = 296 K
0.40 × 0.30 × 0.20 mm

#### Data collection

Rigaku R-AXIS RAPID diffractometer
Absorption correction: multi-scan (*ABSCOR*; Rigaku, 1995 ▶) *T*_min_ = 0.793, *T*_max_ = 0.984
39377 measured reflections
5084 independent reflections
2954 reflections with *F* ^2^ > 2σ(*F* ^2^)
*R*_int_ = 0.037

#### Refinement

*R*[*F* ^2^ > 2σ(*F* ^2^)] = 0.065
*wR*(*F* ^2^) = 0.231
*S* = 1.11
5084 reflections
280 parameters
256 restraints
H-atom parameters constrained
Δρ_max_ = 0.32 e Å^−3^
Δρ_min_ = −0.42 e Å^−3^

### 

Data collection: *RAPID-AUTO* (Rigaku, 2006 ▶); cell refinement: *RAPID-AUTO*; data reduction: *RAPID-AUTO*; program(s) used to solve structure: *SHELXS97* (Sheldrick, 2008 ▶); program(s) used to refine structure: *SHELXL97* (Sheldrick, 2008 ▶); molecular graphics: *CrystalStructure* (Rigaku, 2010 ▶); software used to prepare material for publication: *CrystalStructure*.

## Supplementary Material

Crystal structure: contains datablock(s) global, I. DOI: 10.1107/S1600536811048355/cv5195sup1.cif

Structure factors: contains datablock(s) I. DOI: 10.1107/S1600536811048355/cv5195Isup2.hkl

Additional supplementary materials:  crystallographic information; 3D view; checkCIF report

## Figures and Tables

**Table 1:** Hydrogen-bond geometry (Å, °)

*D*—H⋯*A*	*D*—H	H⋯*A*	*D*⋯*A*	*D*—H⋯*A*
O2—H2*A*⋯O4	0.82	1.82	2.616 (4)	164
O3—H3*A*⋯O1	0.82	1.77	2.572 (4)	164

**Fig. 1.**
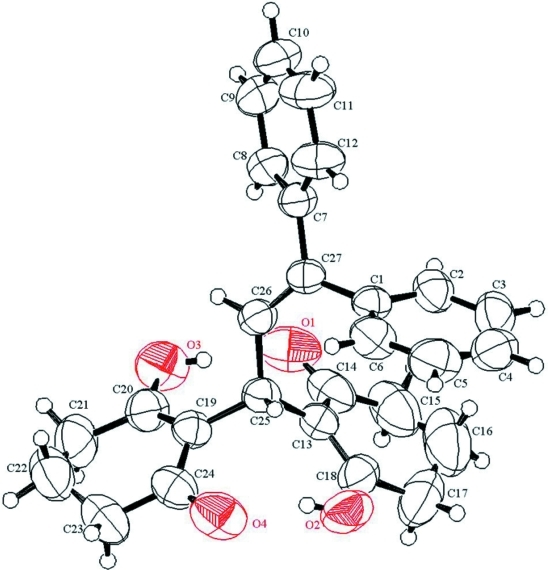
The molecular structure of (I) showing the atomic numbering and 50% probability displacement ellipsoid.

## supplementary crystallographic information

### Comment

As a part of our ongoing study of the substituent effect on the solid state structures of Xanthene derivatives (Cha *et al.*, (2011**a*,b*), we present here the crystal structure of the title compound (I).

In (I) (Fig. 1), the dihedral angle between the two phenyl rings (C1–C6 and C7–C12) is 89.53 (5)°, and the mean planes of two cyclohexenone rings form a dihedral angle of 46.85 (4)°. Both cyclohexenone rings (Fig.1) display half-chair conformation. The hydroxy and carbonyl O atoms face each other and are orientated to allow for the formation of two intramolecular O—H···O hydrogen bonds (Table 1), which are typical for xanthene derivatives.

### Experimental

To a solution of 1,3-cyclohexanedione (4.61 mmol), 2-phenylcinnamaldehyde (1.84 mmol) and 4Å MS was added catalytic amounts of *L*-proline (0.47 mmol) in under nitrogen atmosphere. After stirring for 5 h, The anhydrous ethyl acetate (0.5 ml) was added to a reaction mixture and the solution was stirred for 3 days. The reaction mixture was filtered through pad of celite to remove MS and concentrated. The residue oil was purified by flash column chromatography to afford product which was recrystallized from ethanol to give crystals suitable for X-ray analysis.

### Refinement

C-bound hydrogen atoms were positioned geometrically and refined using a riding model with C—H = 0.93–0.98 Å and *U*iso(H) = 1.2 or 1.5 *U*eq(C). Rotating group model was applied for the methyl groups. The hydroxy H-atom was located in a difference Fourier map, and was isotropically refined with a distance restraint of O—H of 0.82 (1) Å.

### Crystal data

**Table:**

C_27_H_26_O_4_	*F*(000) = 1760.00
*M**_r_* = 414.50	*D*_x_ = 1.233 Mg m^−^^3^
Orthorhombic, *P**b**c**a*	Mo *K*α radiation, λ = 0.71075 Å
Hall symbol: -P 2ac 2ab	Cell parameters from 22114 reflections
*a* = 9.7329 (5) Å	θ = 3.0–27.4°
*b* = 18.6106 (9) Å	µ = 0.08 mm^−^^1^
*c* = 24.6522 (12) Å	*T* = 296 K
*V* = 4465.4 (4) Å^3^	Chunk, colorless
*Z* = 8	0.40 × 0.30 × 0.20 mm

### Data collection

**Table:**

Rigaku R-AXIS RAPID diffractometer	2954 reflections with *F*^2^ > 2σ(*F*^2^)
Detector resolution: 10.000 pixels mm^-1^	*R*_int_ = 0.037
ω scans	θ_max_ = 27.4°
Absorption correction: multi-scan (*ABSCOR*; Rigaku, 1995)	*h* = −12→12
*T*_min_ = 0.793, *T*_max_ = 0.984	*k* = −23→24
39377 measured reflections	*l* = −31→31
5084 independent reflections	

### Refinement

**Table:**

Refinement on *F*^2^	Secondary atom site location: difference Fourier map
*R*[*F*^2^ > 2σ(*F*^2^)] = 0.065	Hydrogen site location: inferred from neighbouring sites
*wR*(*F*^2^) = 0.231	H-atom parameters constrained
*S* = 1.11	*w* = 1/[σ^2^(*F*_o_^2^) + (0.1198*P*)^2^ + 0.5762*P*] where *P* = (*F*_o_^2^ + 2*F*_c_^2^)/3
5084 reflections	(Δ/σ)_max_ < 0.001
280 parameters	Δρ_max_ = 0.32 e Å^−^^3^
256 restraints	Δρ_min_ = −0.42 e Å^−^^3^
Primary atom site location: structure-invariant direct methods	

### Special details

**Table:**

Geometry. All e.s.d.'s (except the e.s.d. in the dihedral angle between two l.s. planes) are estimated using the full covariance matrix. The cell e.s.d.'s are taken into account individually in the estimation of e.s.d.'s in distances, angles and torsion angles; correlations between e.s.d.'s in cell parameters are only used when they are defined by crystal symmetry. An approximate (isotropic) treatment of cell e.s.d.'s is used for estimating e.s.d.'s involving l.s. planes.
Refinement. Refinement was performed using all reflections. The weighted *R*-factor (*wR*) and goodness of fit (*S*) are based on *F*^2^. *R*-factor (gt) are based on *F*. The threshold expression of *F*^2^ > 2.0 σ(*F*^2^) is used only for calculating *R*-factor (gt).

### Fractional atomic coordinates and isotropic or equivalent isotropic displacement parameters (Å^2^)

**Table:**

	*x*	*y*	*z*	*U*_iso_*/*U*_eq_	
O1	0.3447 (3)	0.12993 (17)	0.59891 (10)	0.1037 (9)	
O2	0.4623 (4)	−0.03776 (13)	0.73273 (12)	0.1042 (9)	
O3	0.4111 (3)	0.23227 (14)	0.66394 (11)	0.1008 (9)	
O4	0.5611 (3)	0.06587 (16)	0.79289 (10)	0.1021 (9)	
C1	0.7090 (3)	0.00329 (14)	0.59987 (10)	0.0554 (7)	
C2	0.6468 (4)	−0.03816 (17)	0.56032 (14)	0.0722 (8)	
C3	0.6552 (4)	−0.1130 (2)	0.56267 (18)	0.0927 (10)	
C4	0.7271 (5)	−0.1457 (2)	0.60269 (18)	0.0990 (11)	
C5	0.7898 (5)	−0.1058 (3)	0.64118 (17)	0.0999 (11)	
C6	0.7814 (4)	−0.03125 (18)	0.64031 (13)	0.0779 (9)	
C7	0.7742 (3)	0.11899 (14)	0.55144 (11)	0.0585 (7)	
C8	0.7184 (4)	0.17727 (15)	0.52409 (12)	0.0734 (8)	
C9	0.7915 (5)	0.21151 (18)	0.48286 (14)	0.0883 (10)	
C10	0.9201 (5)	0.1874 (2)	0.46851 (15)	0.0948 (11)	
C11	0.9762 (4)	0.1296 (3)	0.49499 (16)	0.0968 (11)	
C12	0.9032 (4)	0.0949 (2)	0.53547 (13)	0.0812 (9)	
C13	0.4274 (3)	0.04562 (15)	0.66258 (11)	0.0623 (7)	
C14	0.3404 (4)	0.0670 (3)	0.61918 (14)	0.0844 (9)	
C15	0.2377 (5)	0.0162 (4)	0.59619 (18)	0.1210 (13)	
C16	0.2609 (7)	−0.0579 (4)	0.6106 (3)	0.1514 (17)	
C17	0.2893 (5)	−0.0685 (3)	0.6689 (3)	0.1237 (13)	
C18	0.3990 (4)	−0.01717 (17)	0.68890 (17)	0.0847 (9)	
C19	0.5128 (3)	0.14829 (15)	0.72344 (11)	0.0585 (7)	
C20	0.4536 (4)	0.21398 (16)	0.71147 (14)	0.0757 (8)	
C21	0.4332 (5)	0.2703 (2)	0.75493 (19)	0.1023 (11)	
C22	0.5221 (5)	0.2585 (3)	0.8042 (2)	0.1110 (12)	
C23	0.5133 (5)	0.1835 (3)	0.82282 (15)	0.0975 (11)	
C24	0.5313 (4)	0.13004 (19)	0.77861 (13)	0.0748 (8)	
C25	0.5481 (3)	0.09241 (13)	0.68042 (10)	0.0556 (6)	
C26	0.6300 (3)	0.12232 (14)	0.63315 (11)	0.0588 (7)	
C27	0.7002 (3)	0.08350 (14)	0.59723 (10)	0.0533 (6)	
H2	0.5993	−0.0162	0.5321	0.0867*	
H2A	0.5016	−0.0033	0.7465	0.1251*	
H3	0.6111	−0.1406	0.5365	0.1112*	
H3A	0.4045	0.1964	0.6448	0.1210*	
H4	0.7334	−0.1955	0.6037	0.1188*	
H5	0.8391	−0.1284	0.6686	0.1198*	
H6	0.8249	−0.0045	0.6672	0.0934*	
H8	0.6313	0.1937	0.5333	0.0881*	
H9	0.7533	0.2507	0.4650	0.1060*	
H10	0.9688	0.2102	0.4410	0.1138*	
H11	1.0636	0.1137	0.4857	0.1162*	
H12	0.9411	0.0548	0.5522	0.0975*	
H15A	0.1469	0.0302	0.6086	0.1452*	
H15B	0.2387	0.0204	0.5570	0.1452*	
H16A	0.3380	−0.0760	0.5898	0.1817*	
H16B	0.1805	−0.0858	0.6008	0.1817*	
H17A	0.3193	−0.1176	0.6749	0.1485*	
H17B	0.2055	−0.0611	0.6895	0.1485*	
H21A	0.4536	0.3172	0.7398	0.1228*	
H21B	0.3376	0.2703	0.7660	0.1228*	
H22A	0.6168	0.2699	0.7954	0.1332*	
H22B	0.4928	0.2904	0.8331	0.1332*	
H23A	0.4244	0.1760	0.8397	0.1170*	
H23B	0.5831	0.1755	0.8502	0.1170*	
H25	0.6112	0.0592	0.6986	0.0667*	
H26	0.6313	0.1720	0.6289	0.0705*	

### Atomic displacement parameters (Å^2^)

**Table:**

	*U*^11^	*U*^22^	*U*^33^	*U*^12^	*U*^13^	*U*^23^
O1	0.0898 (18)	0.144 (3)	0.0776 (16)	0.0116 (16)	−0.0078 (13)	0.0304 (15)
O2	0.128 (3)	0.0688 (14)	0.116 (2)	0.0128 (14)	0.0350 (17)	0.0296 (13)
O3	0.121 (2)	0.0856 (16)	0.0962 (17)	0.0352 (14)	0.0053 (15)	0.0228 (13)
O4	0.124 (2)	0.1130 (19)	0.0692 (14)	0.0320 (16)	−0.0050 (14)	0.0242 (13)
C1	0.0512 (14)	0.0608 (14)	0.0543 (13)	0.0046 (11)	0.0098 (11)	0.0040 (11)
C2	0.0661 (17)	0.0759 (17)	0.0747 (17)	0.0026 (14)	0.0053 (14)	−0.0115 (14)
C3	0.091 (3)	0.0774 (19)	0.109 (3)	−0.0065 (17)	0.0249 (19)	−0.0282 (18)
C4	0.115 (3)	0.0672 (19)	0.114 (3)	0.0118 (18)	0.050 (3)	0.0060 (17)
C5	0.122 (3)	0.085 (2)	0.093 (3)	0.035 (2)	0.020 (2)	0.0243 (18)
C6	0.089 (2)	0.0776 (18)	0.0669 (17)	0.0216 (16)	−0.0011 (15)	0.0072 (14)
C7	0.0601 (15)	0.0628 (14)	0.0527 (14)	−0.0097 (12)	0.0039 (12)	0.0023 (11)
C8	0.095 (2)	0.0590 (15)	0.0663 (17)	−0.0012 (14)	0.0127 (15)	0.0050 (13)
C9	0.127 (3)	0.0663 (17)	0.0715 (18)	−0.0156 (18)	0.0096 (19)	0.0097 (15)
C10	0.109 (3)	0.098 (3)	0.078 (2)	−0.0418 (19)	0.0207 (19)	0.0051 (17)
C11	0.073 (2)	0.130 (3)	0.087 (3)	−0.0221 (19)	0.0222 (17)	0.015 (2)
C12	0.0604 (17)	0.108 (3)	0.0753 (19)	−0.0035 (16)	0.0106 (15)	0.0189 (17)
C13	0.0622 (15)	0.0632 (14)	0.0616 (14)	0.0007 (12)	0.0159 (12)	−0.0061 (12)
C14	0.0632 (17)	0.125 (3)	0.0653 (17)	−0.0046 (17)	0.0066 (14)	−0.0188 (17)
C15	0.089 (3)	0.172 (3)	0.102 (3)	−0.021 (3)	0.003 (2)	−0.049 (3)
C16	0.115 (3)	0.156 (3)	0.183 (4)	−0.032 (3)	0.016 (3)	−0.080 (4)
C17	0.102 (3)	0.077 (2)	0.192 (4)	−0.0203 (19)	0.038 (3)	−0.022 (3)
C18	0.088 (2)	0.0587 (15)	0.107 (3)	−0.0004 (14)	0.0357 (17)	−0.0062 (15)
C19	0.0543 (14)	0.0617 (14)	0.0595 (14)	0.0039 (11)	0.0040 (11)	−0.0001 (11)
C20	0.0781 (18)	0.0635 (15)	0.0855 (18)	0.0118 (14)	0.0137 (16)	0.0040 (14)
C21	0.104 (3)	0.0751 (19)	0.127 (3)	0.0137 (19)	0.021 (2)	−0.0224 (19)
C22	0.096 (3)	0.119 (3)	0.118 (3)	−0.011 (3)	0.022 (2)	−0.048 (3)
C23	0.087 (3)	0.128 (3)	0.078 (2)	0.002 (2)	0.0019 (18)	−0.0303 (19)
C24	0.0682 (17)	0.0945 (19)	0.0617 (15)	0.0056 (15)	−0.0025 (14)	−0.0035 (14)
C25	0.0572 (14)	0.0559 (13)	0.0536 (13)	0.0053 (11)	0.0061 (11)	0.0041 (11)
C26	0.0618 (15)	0.0537 (13)	0.0609 (15)	−0.0004 (12)	0.0064 (13)	0.0056 (12)
C27	0.0470 (13)	0.0602 (15)	0.0527 (14)	−0.0012 (11)	0.0017 (11)	0.0039 (12)

### Geometric parameters (Å, °)

**Table:**

O1—C14	1.274 (6)	C22—C23	1.471 (7)
O2—C18	1.301 (5)	C23—C24	1.487 (6)
O3—C20	1.289 (5)	C25—C26	1.518 (4)
O4—C24	1.278 (5)	C26—C27	1.332 (4)
C1—C2	1.383 (5)	O2—H2A	0.820
C1—C6	1.380 (5)	O3—H3A	0.820
C1—C27	1.497 (4)	C2—H2	0.930
C2—C3	1.396 (5)	C3—H3	0.930
C3—C4	1.354 (6)	C4—H4	0.930
C4—C5	1.350 (6)	C5—H5	0.930
C5—C6	1.389 (6)	C6—H6	0.930
C7—C8	1.388 (4)	C8—H8	0.930
C7—C12	1.389 (5)	C9—H9	0.930
C7—C27	1.493 (4)	C10—H10	0.930
C8—C9	1.395 (5)	C11—H11	0.930
C9—C10	1.375 (6)	C12—H12	0.930
C10—C11	1.371 (6)	C15—H15A	0.970
C11—C12	1.385 (6)	C15—H15B	0.970
C13—C14	1.421 (5)	C16—H16A	0.970
C13—C18	1.365 (5)	C16—H16B	0.970
C13—C25	1.527 (4)	C17—H17A	0.970
C14—C15	1.488 (7)	C17—H17B	0.970
C15—C16	1.442 (9)	C21—H21A	0.970
C16—C17	1.476 (10)	C21—H21B	0.970
C17—C18	1.516 (6)	C22—H22A	0.970
C19—C20	1.383 (5)	C22—H22B	0.970
C19—C24	1.413 (5)	C23—H23A	0.970
C19—C25	1.525 (4)	C23—H23B	0.970
C20—C21	1.512 (6)	C25—H25	0.980
C21—C22	1.507 (7)	C26—H26	0.930
			
O1···O3	2.572 (4)	O4···H17B^iii^	2.7827
O1···C18	3.563 (5)	C2···H2^v^	3.4576
O1···C19	3.495 (4)	C2···H11^ix^	3.3475
O1···C20	3.357 (5)	C2···H15B^v^	3.1161
O1···C25	2.906 (4)	C3···H11^ix^	2.9851
O1···C26	2.906 (4)	C3···H15B^v^	3.5681
O1···C27	3.567 (4)	C3···H22B^vi^	3.4522
O2···O4	2.616 (4)	C4···H10^ix^	3.3705
O2···C19	3.505 (4)	C4···H11^ix^	3.0416
O2···C24	3.389 (5)	C4···H22B^vi^	2.9167
O2···C25	2.869 (4)	C5···H11^ix^	3.4416
O3···C13	3.477 (4)	C5···H21B^vi^	3.4779
O3···C14	3.340 (5)	C5···H22B^vi^	3.4210
O3···C25	2.953 (4)	C6···H2A^iii^	3.5575
O3···C26	3.050 (4)	C7···H23A^iii^	3.2349
O4···C13	3.487 (4)	C8···H4^vii^	3.1103
O4···C18	3.384 (5)	C8···H10^ii^	3.3210
O4···C20	3.567 (5)	C8···H16A^v^	3.4258
O4···C25	2.819 (4)	C9···H3^vii^	3.1973
C1···C4	2.779 (5)	C9···H4^vii^	3.4531
C1···C12	2.999 (5)	C9···H16A^v^	3.3402
C1···C13	3.244 (4)	C9···H16B^v^	3.1302
C1···C25	3.024 (4)	C10···H3A^iv^	3.5357
C2···C5	2.737 (6)	C10···H8^iv^	3.0203
C2···C7	3.184 (4)	C10···H16A^v^	3.5602
C2···C12	3.568 (5)	C10···H16B^v^	2.7300
C2···C26	3.489 (5)	C11···H16B^v^	2.9261
C3···C6	2.736 (6)	C12···H15A^x^	3.2138
C6···C7	3.553 (5)	C12···H15B^x^	3.5876
C6···C25	3.381 (5)	C12···H15B^v^	3.4213
C6···C26	3.220 (5)	C12···H16B^v^	3.4596
C7···C10	2.796 (5)	C12···H23A^iii^	3.4340
C8···C11	2.755 (6)	C14···H23B^i^	3.3039
C8···C26	3.002 (4)	C15···H2^v^	3.5388
C9···C12	2.752 (5)	C15···H12^xi^	3.1655
C13···C16	2.824 (7)	C15···H23B^i^	3.5767
C13···C20	3.367 (5)	C17···H22A^vi^	3.2641
C13···C24	3.417 (5)	C17···H22B^vi^	3.3764
C13···C27	3.185 (4)	C20···H22A^i^	3.4440
C14···C17	2.848 (7)	C21···H22A^i^	3.3203
C14···C19	3.422 (5)	C22···H4^xii^	3.4753
C14···C26	3.021 (5)	C22···H17A^xii^	2.8229
C14···C27	3.557 (5)	C22···H21B^iii^	3.5308
C15···C18	2.842 (6)	C23···H15A^iii^	3.5630
C18···C19	3.381 (5)	C24···H6^i^	3.4777
C19···C22	2.859 (6)	C24···H15A^iii^	3.5282
C20···C23	2.862 (5)	C26···H23A^iii^	3.1072
C20···C26	3.096 (5)	C27···H23A^iii^	3.1847
C21···C24	2.841 (6)	H2···C2^v^	3.4576
O2···C6^i^	3.593 (5)	H2···C15^v^	3.5388
O3···C10^ii^	3.592 (5)	H2···H2^v^	2.5705
O4···C14^iii^	3.477 (5)	H2···H15B^v^	2.7044
O4···C15^iii^	3.359 (6)	H2···H16A^v^	3.5143
O4···C17^iii^	3.475 (6)	H2A···C6^i^	3.5575
C6···O2^iii^	3.593 (5)	H2A···H5^i^	3.5083
C10···O3^iv^	3.592 (5)	H2A···H6^i^	2.7375
C10···C16^v^	3.567 (8)	H2A···H17B^iii^	2.7549
C14···O4^i^	3.477 (5)	H2A···H21A^vi^	3.3852
C15···O4^i^	3.359 (6)	H3···O1^v^	3.3721
C16···C10^v^	3.567 (8)	H3···C9^xiii^	3.1973
C17···O4^i^	3.475 (6)	H3···H8^v^	3.0832
O1···H3A	1.7743	H3···H9^xiii^	2.9899
O1···H8	3.4351	H3···H11^ix^	3.2522
O1···H15A	2.6845	H3···H15B^v^	3.5288
O1···H15B	2.5074	H3A···C10^ii^	3.5357
O1···H26	2.9899	H3A···H9^ii^	3.2346
O2···H17A	2.4854	H3A···H10^ii^	2.8081
O2···H17B	2.7518	H3A···H22A^i^	3.4478
O2···H25	2.4622	H3A···H23B^i^	3.1539
O3···H21A	2.4836	H4···C8^xiii^	3.1103
O3···H21B	2.7095	H4···C9^xiii^	3.4531
O3···H26	2.5690	H4···C22^vi^	3.4753
O4···H2A	1.8174	H4···H8^xiii^	2.9986
O4···H23A	2.7028	H4···H9^xiii^	3.5640
O4···H23B	2.4906	H4···H10^ix^	3.1126
O4···H25	2.3782	H4···H11^ix^	3.3286
C1···H3	3.2435	H4···H21B^vi^	3.3474
C1···H5	3.2374	H4···H22B^vi^	2.7103
C1···H12	2.7198	H4···H23A^vi^	3.1659
C1···H25	2.8128	H4···H26^xiii^	2.8640
C1···H26	3.3075	H5···O2^iii^	3.1939
C2···H4	3.2294	H5···O3^xiii^	3.5566
C2···H6	3.2144	H5···H2A^iii^	3.5083
C2···H12	3.3514	H5···H21A^xiii^	2.8593
C2···H16A	3.1720	H5···H21B^vi^	3.0184
C3···H5	3.1787	H5···H22B^vi^	3.5664
C3···H16A	3.2335	H6···O2^iii^	2.8745
C4···H2	3.2224	H6···O4^iii^	2.8235
C4···H6	3.2147	H6···C24^iii^	3.4777
C5···H3	3.1786	H6···H2A^iii^	2.7375
C6···H2	3.2145	H6···H15A^x^	3.5109
C6···H4	3.2218	H6···H23A^iii^	3.5004
C6···H12	3.1131	H8···C10^ii^	3.0203
C6···H25	2.7642	H8···H3^v^	3.0832
C7···H2	3.0743	H8···H4^vii^	2.9986
C7···H9	3.2545	H8···H10^ii^	2.4697
C7···H11	3.2507	H9···O1^iv^	2.8650
C7···H26	2.5603	H9···O3^iv^	3.5448
C8···H10	3.2420	H9···H3^vii^	2.9899
C8···H12	3.2211	H9···H3A^iv^	3.2346
C8···H26	2.7215	H9···H4^vii^	3.5640
C9···H11	3.2142	H9···H11^ii^	3.3539
C10···H8	3.2358	H9···H16B^v^	3.5312
C10···H12	3.2245	H9···H23B^xiv^	3.5553
C11···H9	3.2140	H10···O1^iv^	3.3580
C12···H8	3.2226	H10···O3^iv^	2.8553
C12···H10	3.2311	H10···C4^ix^	3.3705
C13···H2A	2.3721	H10···C8^iv^	3.3210
C13···H3A	2.8489	H10···H3A^iv^	2.8081
C13···H15A	3.0501	H10···H4^ix^	3.1126
C13···H15B	3.2200	H10···H8^iv^	2.4697
C13···H16A	3.0166	H10···H16B^v^	2.9215
C13···H17A	3.2289	H10···H26^iv^	3.2059
C13···H17B	3.0071	H11···C2^ix^	3.3475
C13···H26	3.1871	H11···C3^ix^	2.9851
C14···H3A	2.5669	H11···C4^ix^	3.0416
C14···H16A	2.7581	H11···C5^ix^	3.4416
C14···H16B	3.2724	H11···H3^ix^	3.2522
C14···H17B	3.2252	H11···H4^ix^	3.3286
C14···H25	3.2866	H11···H9^iv^	3.3539
C14···H26	3.4486	H11···H12^ix^	3.2720
C15···H17A	3.2556	H11···H15A^x^	3.4997
C15···H17B	2.7298	H11···H15B^x^	3.0007
C17···H2A	3.0662	H11···H16B^v^	3.2342
C17···H15A	2.7398	H12···C15^x^	3.1655
C17···H15B	3.2551	H12···H11^ix^	3.2720
C18···H15A	3.2736	H12···H12^ix^	3.4791
C18···H16A	2.7422	H12···H15A^x^	2.4806
C18···H16B	3.2978	H12···H15B^x^	2.9692
C18···H25	2.5179	H12···H15B^v^	3.5027
C19···H2A	2.8798	H12···H23A^iii^	3.4951
C19···H3A	2.3816	H12···H23B^iii^	3.5698
C19···H21A	3.2220	H15A···O4^i^	2.6524
C19···H21B	3.0271	H15A···C12^xi^	3.2138
C19···H22A	3.0478	H15A···C23^i^	3.5630
C19···H23A	3.0366	H15A···C24^i^	3.5282
C19···H23B	3.2388	H15A···H6^xi^	3.5109
C19···H26	2.6372	H15A···H11^xi^	3.4997
C20···H22A	2.8074	H15A···H12^xi^	2.4806
C20···H22B	3.3394	H15A···H23B^i^	2.9540
C20···H23A	3.2516	H15B···C2^v^	3.1161
C20···H25	3.2792	H15B···C3^v^	3.5681
C20···H26	2.7828	H15B···C12^xi^	3.5876
C21···H3A	3.0564	H15B···C12^v^	3.4213
C21···H23A	2.7303	H15B···H2^v^	2.7044
C21···H23B	3.2804	H15B···H3^v^	3.5288
C23···H21A	3.2738	H15B···H11^xi^	3.0007
C23···H21B	2.7372	H15B···H12^xi^	2.9692
C24···H2A	2.6211	H15B···H12^v^	3.5027
C24···H21B	3.2351	H16A···C8^v^	3.4258
C24···H22A	2.7633	H16A···C9^v^	3.3402
C24···H22B	3.2931	H16A···C10^v^	3.5602
C24···H25	2.4968	H16A···H2^v^	3.5143
C25···H2A	2.4552	H16A···H22B^vi^	3.5374
C25···H3A	2.5440	H16B···C9^v^	3.1302
C25···H6	3.2579	H16B···C10^v^	2.7300
C26···H2	3.5965	H16B···C11^v^	2.9261
C26···H3A	2.6084	H16B···C12^v^	3.4596
C26···H6	3.1414	H16B···H9^v^	3.5312
C26···H8	2.7960	H16B···H10^v^	2.9215
C27···H2	2.6422	H16B···H11^v^	3.2342
C27···H6	2.6692	H17A···O3^xv^	3.5925
C27···H8	2.6713	H17A···C22^vi^	2.8229
C27···H12	2.6478	H17A···H21A^xv^	3.3290
C27···H25	2.6835	H17A···H21A^vi^	3.2834
H2···H3	2.3214	H17A···H21B^xv^	3.4245
H2···H16A	3.1199	H17A···H22A^vi^	2.3041
H2A···H17A	3.2842	H17A···H22B^vi^	2.5139
H2A···H17B	3.3817	H17B···O2^i^	3.0777
H2A···H25	1.9701	H17B···O4^i^	2.7827
H3···H4	2.2811	H17B···H2A^i^	2.7549
H3···H16A	3.1993	H17B···H21A^xv^	3.0113
H3A···H8	3.5249	H21A···O2^xii^	2.9003
H3A···H21A	3.2817	H21A···H2A^xii^	3.3852
H3A···H21B	3.3526	H21A···H5^vii^	2.8593
H3A···H25	3.5114	H21A···H17A^viii^	3.3290
H3A···H26	2.2879	H21A···H17A^xii^	3.2834
H4···H5	2.2758	H21A···H17B^viii^	3.0113
H5···H6	2.3106	H21A···H22A^i^	3.5035
H6···H12	3.2436	H21B···C5^xii^	3.4779
H6···H25	2.5159	H21B···C22^i^	3.5308
H8···H9	2.3182	H21B···H4^xii^	3.3474
H8···H26	2.3902	H21B···H5^xii^	3.0184
H9···H10	2.3064	H21B···H17A^viii^	3.4245
H10···H11	2.3011	H21B···H22A^i^	2.6281
H11···H12	2.3053	H22A···O3^iii^	3.1148
H15A···H16A	2.7532	H22A···C17^xii^	3.2641
H15A···H16B	2.1916	H22A···C20^iii^	3.4440
H15A···H17B	2.6799	H22A···C21^iii^	3.3203
H15B···H16A	2.1929	H22A···H3A^iii^	3.4478
H15B···H16B	2.3219	H22A···H17A^xii^	2.3041
H16A···H17A	2.2432	H22A···H21A^iii^	3.5035
H16A···H17B	2.7881	H22A···H21B^iii^	2.6281
H16B···H17A	2.3488	H22B···C3^xii^	3.4522
H16B···H17B	2.2477	H22B···C4^xii^	2.9167
H21A···H22A	2.2751	H22B···C5^xii^	3.4210
H21A···H22B	2.3841	H22B···C17^xii^	3.3764
H21B···H22A	2.8122	H22B···H4^xii^	2.7103
H21B···H22B	2.2707	H22B···H5^xii^	3.5664
H21B···H23A	2.6634	H22B···H16A^xii^	3.5374
H22A···H23A	2.7841	H22B···H17A^xii^	2.5139
H22A···H23B	2.2408	H23A···C7^i^	3.2349
H22B···H23A	2.2353	H23A···C12^i^	3.4340
H22B···H23B	2.3500	H23A···C26^i^	3.1072
H25···H26	2.7197	H23A···C27^i^	3.1847
O1···H3^v^	3.3721	H23A···H4^xii^	3.1659
O1···H9^ii^	2.8650	H23A···H6^i^	3.5004
O1···H10^ii^	3.3580	H23A···H12^i^	3.4951
O1···H23B^i^	2.9620	H23A···H26^i^	2.9568
O2···H5^i^	3.1939	H23B···O1^iii^	2.9620
O2···H6^i^	2.8745	H23B···O3^iii^	3.3802
O2···H17B^iii^	3.0777	H23B···C14^iii^	3.3039
O2···H21A^vi^	2.9003	H23B···C15^iii^	3.5767
O3···H5^vii^	3.5566	H23B···H3A^iii^	3.1539
O3···H9^ii^	3.5448	H23B···H9^xvi^	3.5553
O3···H10^ii^	2.8553	H23B···H12^i^	3.5698
O3···H17A^viii^	3.5925	H23B···H15A^iii^	2.9540
O3···H22A^i^	3.1148	H26···H4^vii^	2.8640
O3···H23B^i^	3.3802	H26···H10^ii^	3.2059
O4···H6^i^	2.8235	H26···H23A^iii^	2.9568
O4···H15A^iii^	2.6524		
			
C2—C1—C6	118.2 (3)	C4—C3—H3	119.711
C2—C1—C27	120.1 (3)	C3—C4—H4	120.074
C6—C1—C27	121.7 (3)	C5—C4—H4	120.054
C1—C2—C3	120.1 (4)	C4—C5—H5	119.609
C2—C3—C4	120.6 (4)	C6—C5—H5	119.607
C3—C4—C5	119.9 (4)	C1—C6—H6	119.803
C4—C5—C6	120.8 (4)	C5—C6—H6	119.787
C1—C6—C5	120.4 (4)	C7—C8—H8	119.634
C8—C7—C12	117.9 (3)	C9—C8—H8	119.606
C8—C7—C27	121.6 (3)	C8—C9—H9	119.923
C12—C7—C27	120.5 (3)	C10—C9—H9	119.904
C7—C8—C9	120.8 (4)	C9—C10—H10	120.164
C8—C9—C10	120.2 (4)	C11—C10—H10	120.158
C9—C10—C11	119.7 (4)	C10—C11—H11	119.834
C10—C11—C12	120.3 (4)	C12—C11—H11	119.832
C7—C12—C11	121.1 (4)	C7—C12—H12	119.449
C14—C13—C18	118.5 (3)	C11—C12—H12	119.447
C14—C13—C25	121.0 (3)	C14—C15—H15A	108.733
C18—C13—C25	120.4 (3)	C14—C15—H15B	108.738
O1—C14—C13	122.2 (4)	C16—C15—H15A	108.748
O1—C14—C15	117.2 (4)	C16—C15—H15B	108.746
C13—C14—C15	120.6 (4)	H15A—C15—H15B	107.626
C14—C15—C16	114.1 (5)	C15—C16—H16A	108.872
C15—C16—C17	113.5 (5)	C15—C16—H16B	108.870
C16—C17—C18	111.3 (5)	C17—C16—H16A	108.872
O2—C18—C13	123.5 (4)	C17—C16—H16B	108.877
O2—C18—C17	114.7 (4)	H16A—C16—H16B	107.718
C13—C18—C17	121.9 (4)	C16—C17—H17A	109.355
C20—C19—C24	118.1 (3)	C16—C17—H17B	109.364
C20—C19—C25	123.3 (3)	C18—C17—H17A	109.367
C24—C19—C25	118.5 (3)	C18—C17—H17B	109.380
O3—C20—C19	124.1 (3)	H17A—C17—H17B	107.993
O3—C20—C21	114.8 (3)	C20—C21—H21A	108.948
C19—C20—C21	121.1 (3)	C20—C21—H21B	108.946
C20—C21—C22	113.2 (4)	C22—C21—H21A	108.929
C21—C22—C23	110.9 (4)	C22—C21—H21B	108.933
C22—C23—C24	113.5 (4)	H21A—C21—H21B	107.750
O4—C24—C19	121.2 (3)	C21—C22—H22A	109.454
O4—C24—C23	116.8 (3)	C21—C22—H22B	109.456
C19—C24—C23	122.0 (3)	C23—C22—H22A	109.464
C13—C25—C19	114.6 (3)	C23—C22—H22B	109.452
C13—C25—C26	113.1 (3)	H22A—C22—H22B	108.047
C19—C25—C26	113.7 (2)	C22—C23—H23A	108.861
C25—C26—C27	125.5 (3)	C22—C23—H23B	108.865
C1—C27—C7	116.5 (3)	C24—C23—H23A	108.863
C1—C27—C26	122.8 (3)	C24—C23—H23B	108.867
C7—C27—C26	120.7 (3)	H23A—C23—H23B	107.718
C18—O2—H2A	109.468	C13—C25—H25	104.699
C20—O3—H3A	109.476	C19—C25—H25	104.698
C1—C2—H2	119.945	C26—C25—H25	104.697
C3—C2—H2	119.954	C25—C26—H26	117.235
C2—C3—H3	119.715	C27—C26—H26	117.232
			
C2—C1—C6—C5	−0.6 (5)	C18—C13—C25—C19	−89.8 (4)
C6—C1—C2—C3	1.6 (5)	C18—C13—C25—C26	137.7 (3)
C2—C1—C27—C7	−67.3 (4)	C25—C13—C18—O2	6.5 (5)
C2—C1—C27—C26	112.3 (3)	C25—C13—C18—C17	−173.8 (3)
C27—C1—C2—C3	179.8 (3)	O1—C14—C15—C16	165.0 (3)
C6—C1—C27—C7	110.9 (3)	C13—C14—C15—C16	−16.2 (5)
C6—C1—C27—C26	−69.5 (4)	C14—C15—C16—C17	46.8 (6)
C27—C1—C6—C5	−178.9 (3)	C15—C16—C17—C18	−49.3 (6)
C1—C2—C3—C4	−1.8 (6)	C16—C17—C18—O2	−157.9 (4)
C2—C3—C4—C5	1.0 (7)	C16—C17—C18—C13	22.4 (6)
C3—C4—C5—C6	0.0 (7)	C20—C19—C24—O4	166.9 (3)
C4—C5—C6—C1	−0.2 (7)	C20—C19—C24—C23	−11.3 (5)
C8—C7—C12—C11	2.5 (5)	C24—C19—C20—O3	−168.4 (3)
C12—C7—C8—C9	−1.6 (5)	C24—C19—C20—C21	11.1 (5)
C8—C7—C27—C1	140.6 (3)	C20—C19—C25—C13	−82.0 (4)
C8—C7—C27—C26	−39.0 (4)	C20—C19—C25—C26	50.2 (4)
C27—C7—C8—C9	177.4 (3)	C25—C19—C20—O3	6.5 (5)
C12—C7—C27—C1	−40.4 (4)	C25—C19—C20—C21	−174.0 (3)
C12—C7—C27—C26	140.0 (3)	C24—C19—C25—C13	92.9 (3)
C27—C7—C12—C11	−176.5 (3)	C24—C19—C25—C26	−134.9 (3)
C7—C8—C9—C10	0.5 (5)	C25—C19—C24—O4	−8.3 (5)
C8—C9—C10—C11	−0.1 (6)	C25—C19—C24—C23	173.6 (3)
C9—C10—C11—C12	1.0 (6)	O3—C20—C21—C22	−161.3 (3)
C10—C11—C12—C7	−2.2 (6)	C19—C20—C21—C22	19.1 (5)
C14—C13—C18—O2	−172.1 (3)	C20—C21—C22—C23	−48.5 (5)
C14—C13—C18—C17	7.6 (5)	C21—C22—C23—C24	48.6 (5)
C18—C13—C14—O1	167.5 (3)	C22—C23—C24—O4	162.1 (3)
C18—C13—C14—C15	−11.3 (5)	C22—C23—C24—C19	−19.7 (5)
C14—C13—C25—C19	88.8 (3)	C13—C25—C26—C27	−63.5 (4)
C14—C13—C25—C26	−43.7 (4)	C19—C25—C26—C27	163.6 (3)
C25—C13—C14—O1	−11.2 (5)	C25—C26—C27—C1	−1.2 (4)
C25—C13—C14—C15	170.0 (3)	C25—C26—C27—C7	178.4 (3)

Symmetry codes: (i) *x*−1/2, *y*, −*z*+3/2; (ii) *x*−1/2, −*y*+1/2, −*z*+1; (iii) *x*+1/2, *y*, −*z*+3/2; (iv) *x*+1/2, −*y*+1/2, −*z*+1; (v) −*x*+1, −*y*, −*z*+1; (vi) −*x*+1, *y*−1/2, −*z*+3/2; (vii) −*x*+3/2, *y*+1/2, *z*; (viii) −*x*+1/2, *y*+1/2, *z*; (ix) −*x*+2, −*y*, −*z*+1; (x) *x*+1, *y*, *z*; (xi) *x*−1, *y*, *z*; (xii) −*x*+1, *y*+1/2, −*z*+3/2; (xiii) −*x*+3/2, *y*−1/2, *z*; (xiv) *x*, −*y*+1/2, *z*−1/2; (xv) −*x*+1/2, *y*−1/2, *z*; (xvi) *x*, −*y*+1/2, *z*+1/2.

### Hydrogen-bond geometry (Å, °)

**Table:**

*D*—H···*A*	*D*—H	H···*A*	*D*···*A*	*D*—H···*A*
O2—H2A···O4	0.82	1.82	2.616 (4)	164.
O3—H3A···O1	0.82	1.77	2.572 (4)	164.
